# Regionconnect: Rapidly extracting standardized brain connectivity information in voxel-wise neuroimaging studies

**DOI:** 10.1016/j.neuroimage.2020.117462

**Published:** 2020-10-16

**Authors:** Xiaoxiao Qi, Konstantinos Arfanakis

**Affiliations:** a Department of Biomedical Engineering, Illinois Institute of Technology, Chicago, IL, United States; b Rush Alzheimer’s Disease Center, Rush University Medical Center, Chicago, IL, United States; c Department of Diagnostic Radiology and Nuclear Medicine, Rush University Medical Center, Chicago, IL, United States

**Keywords:** Regionconnect, Brain, Connectivity, White matter, Atlas

## Abstract

Reporting white matter findings in voxel-wise neuroimaging studies typically lacks specificity in terms of brain connectivity. Therefore, the purpose of this work was to develop an approach for rapidly extracting standardized brain connectivity information for white matter regions with significant findings in voxel-wise neuroimaging studies. The new approach was named regionconnect and is based on precalculated average healthy adult brain connectivity information stored in standard space in a fashion that allows fast retrieval and integration. Towards this goal, the present work first generated and evaluated the white matter connectome of the IIT Human Brain Atlas v.5.0. It was demonstrated that the edges of the atlas connectome are representative of those of individual participants of the Human Connectome Project in terms of the spatial organization of streamlines and spatial patterns of track-density. Next, the new white matter connectome was used to develop multi-layer, connectivity-based labels for each white matter voxel of the atlas, consistent with the fact that each voxel may contain axons from multiple connections. The regionconnect algorithm was then developed to rapidly integrate information contained in the multi-layer labels across voxels of a white matter region and to generate a list of the most probable connections traversing that region. Usage of regionconnect does not require high angular resolution diffusion MRI or any MRI data. The regionconnect algorithm as well as the white matter tractogram and connectome, multi-layer, connectivity-based labels, and associated resources developed for the IIT Human Brain Atlas v.5.0 in this work are available at www.nitrc.org/projects/iit. An interactive, online version of regionconnect is also available at www.iit.edu/~mri.

## 1. Introduction

Voxel-wise neuroimaging studies typically report white matter findings by simply displaying the resulting statistical maps and/or providing tables of the results for clusters with statistically significant findings (Chang et al., 2012; Chu et al., 2010; Deoni et al., 2015; Focke et al., 2008; Hua et al., 2008; Kim et al., 2011; Scherfler et al., 2006; Smith et al., 2006; Woolley et al., 2011). In some cases, the names of white matter structures that include clusters with significant findings may be provided (e.g. internal capsule, corpus callosum) (Càmara et al., 2007; Duran et al., 2009; Lipton et al., 2008; Xie et al., 2006), and mostly when diffusion MRI data are available the major fiber bundles implicated may also be listed (e.g. superior longitudinal fasciculus, corona radiata) (Choi et al., 2015; Karlsgodt et al., 2008; Lee et al., 2010; Oechslin et al., 2009). However, the above provide little specificity in terms of brain connectivity, which is the main focus when there is a statistically significant finding in white matter. Major white matter structures or fiber bundles contain multiple connections of functionally diverse gray matter regions (Jeurissen et al., 2013; Sporns, 2011), and also the information presented in statistical maps resulting from voxel-wise analyses cannot be easily integrated to assess which connections are implicated. In contrast, gray matter findings of voxel-wise neuroimaging studies are reported in a fashion that is far more specific and informative about the main role of gray matter, namely brain function. This is achieved by simply presenting the name or standard space coordinates of a gray matter region. For example, presenting a finding in the lateral geniculate nucleus of the thalamus implicates vision, and providing standard space coordinates of a gray matter region allows use of a brain function database to extract information about function (Laird et al., 2005; Yarkoni et al., 2011). This level of specificity that is currently feasible in voxel-wise neuroimaging studies for linking gray matter findings to brain function is typically not feasible for linking white matter findings to brain connectivity.

White matter tractography could be added at the end of a voxel-wise investigation to enhance specificity about brain connectivity by using the clusters with significant findings as seeds. However, this requires high-quality diffusion MRI data with sufficiently high angular resolution, as well as a significant time investment for performing tractography for each subject, and spatially normalizing and summarizing the information across subjects. Very often, such data is not available as the research and clinical interests related to white matter are not limited to water diffusion (e.g. a study of white matter may be focused on T_1_, T_2_, myelin water fraction, magnetic susceptibility, or spectroscopic measurements), and in some cases no MRI data may be available (e.g. in studies using positron emission tomography (PET)). Furthermore, tractography may not be appropriate in persons with certain lesions (e.g. infarcts, microbleeds, enlarged perivascular spaces) that may cause it to fail in detecting a connection even though a connection may exist. Finally, differences in the quality of diffusion MRI data, tractography methods, and tractography expertise, may lead to different connectivity results across studies. Overall, enhancing the specificity of white matter findings in voxel-wise analyses with regards to brain connectivity is highly desirable, but tractography using the significant findings as seeds may not be feasible, optimal, efficient, or reproducible.

The purpose of this work was to develop an approach for rapidly extracting standardized brain connectivity information for white matter regions with significant findings in voxel-wise neuroimaging studies. This approach can be outlined as follows: a) healthy brain connectivity information is first precalculated for every white matter voxel in standard space and is stored in a fashion that allows fast retrieval and integration, b) voxel-wise analysis is either conducted in standard space or the results of the analysis are transformed to standard space, and c) standardized brain connectivity information is integrated and summarized rapidly and reproducibly for regions with significant findings, even in the absence of diffusion MRI or any MRI data. Towards this goal, the present work first generated the white matter connectome of the IIT Human Brain Atlas v.5.0 (Zhang and Arfanakis, 2018), then introduced and developed multi-layer, connectivity-based labels for each white matter voxel of the atlas, and finally generated software that integrates the information in the multi-layer labels across voxels of a white matter region to rapidly derive a list of the most probable connections traversing that region. The IIT Human Brain Atlas was selected because it contains structural, diffusion tensor imaging (Zhang and Arfanakis, 2018), and high angular resolution diffusion imaging (HARDI) (Varentsova et al., 2014) templates of the healthy young adult brain, allowing versatile spatial normalization to its space for images collected with various MR and non-MR image contrasts using anatomy-based, tensor-based, or fODF-based (fiber orientation distribution function) registration. In addition, the IIT Human Brain Atlas is attractive for building a white matter connectome since the diffusion-weighted data of the atlas were collected using Turboprop diffusion MRI that is relatively immune to image distortions and artifacts encountered in conventional echo-planar imaging (EPI) (Zhang et al., 2011). The new white matter connectome was used to develop multi-layer, connectivity-based labels for each white matter voxel of the atlas, consistent with the fact that each voxel may contain axons from multiple connections (Jeurissen et al., 2013). The multi-layer labels constituted efficient representations of connectivity information per voxel, allowing software also developed in this work to rapidly integrate information across voxels of a white matter region and generate a list of the most probable connections traversing that region. This software was named regionconnect and is available on www.nitrc.org/projects/iit (an interactive demo version is also available at www.iit.edu/~mri).

## 2. Methods

This section is divided into three sub-sections. The first describes the procedures for constructing and evaluating the white matter connectome of the IIT Human Brain Atlas v.5.0. The second sub-section introduces the concept of multi-layer, connectivity-based white matter labels and describes the process for constructing such labels for each white matter voxel of the IIT Human Brain Atlas v.5.0. The third sub-section presents the development of the regionconnect software which uses the space and multi-layer white matter labels of the IIT Human Brain Atlas v.5.0 to rapidly extract standardized brain connectivity information for white matter regions with significant findings in voxel-wise neuroimaging studies.

### 2.1. White matter connectome of the IIT Human Brain Atlas v.5.0

#### 2.1.1. Available atlas resources used in the development of the white matter connectome

The previously developed HARDI template (Varentsova et al., 2014) and probabilistic gray matter labels of the IIT Human Brain Atlas (Qi et al., 2017) (available at www.nitrc.org/projects/iit) were used in the construction of the white matter connectome. Briefly, the HARDI template was previously constructed using artifact-free Turboprop diffusion imaging data on 72 healthy young adults (42% male, 20–40 years of age). Fiber orientation distribution functions (fODF) were reconstructed in each voxel of the HARDI template using constrained spherical deconvolution (CSD) with up to l_max_=6 order modified real spherical harmonics (SH) basis (Tournier et al., 2004, 2007).

The spatial transformations applied to the artifact-free diffusion data of the 72 healthy young adults for the development of the HARDI template were also applied to the Desikan-Killiany segmentations of the corresponding T_1_-weighted data (Desikan et al., 2006), and the gray matter labels from all subjects in atlas space were used to construct the probabilistic gray matter labels of the atlas. Majority voting was used to convert the probabilistic gray matter labels into deterministic labels. The HARDI template and gray matter labels of the atlas are located in the same space. The voxel-size of all available resources of the IIT Human Brain Atlas v.5.0 is 1 mm × 1 mm × 1 mm.

#### 2.1.2. Available data used for evaluation of the white matter connectome

Pre-processed structural T_1_ -weighted and diffusion MRI data from 20 unrelated young adult participants of the Human Connectome Project (HCP) (50% male, 22–36 years of age) were used to evaluate the white matter connectome of the IIT Human Brain Atlas v.5.0. The diffusion-weighted images were collected using a spin-echo EPI-based sequence that included three shells of *b* = 1000, 2000, and 3000 s/mm^2^, with approximately 90 diffusion weighting directions per shell and a total of 36 *b* = 0 s/mm^2^ volumes. Details on the HCP MRI protocol and preprocessing can be found in (Glasser et al., 2013; Van Essen et al., 2013).

#### 2.1.3. Development of the white matter connectome of the IIT human brain atlas

Probabilistic tractography using the second-order integration over fiber orientation distributions (iFOD2) algorithm (Tournier et al., 2010) was performed on the HARDI template of the IIT Human Brain Atlas in MRtrix3 (Tournier et al., 2012). Anatomically-constrained tractography (ACT) using the gray matter, white matter, and cerebrospinal fluid probability maps of the atlas as priors was employed for streamline termination (Smith et al., 2012). One hundred million streamlines were generated with a step size of 1 mm, dynamic seeding, backtracking, maximum allowed angle of 45°, minimum and maximum streamline length of 2 mm and 250 mm, respectively. The resulting tractogram was filtered to 10 million streamlines using spherical-deconvolution informed filtering of tractograms (SIFT) (Smith et al., 2013) and was made available at www.nitrc.org/projects/iit. The above tractography approach was evaluated using the framework described in Maier-Hein et al., 2017 and the results are provided in Appendix 1.

In order to preserve connections through the optic chiasm, spinal cord, and fornix in the connectome, labels of these three structures were added to the existing gray matter labels of the atlas. The first label was defined in a single coronal slice through the optic chiasm, the second label was positioned in a single axial slice at the level of the medulla, and the third label was defined in a single axial slice through the anterior portion of the fornix just superior of the anterior commissure. Appendix 2 presents the augmented list of gray matter labels used in the development of the IIT connectome. In the following, we refer to this augmented list of labels as “gray matter labels” for simplicity.

The tractogram generated above was combined with the augmented gray matter labels to identify the streamlines connecting the different pairs of gray matter labels, thereby generating the white matter connectome of the IIT Human Brain Atlas v.5.0. A symmetric 88 by 88 connectivity matrix including 3828 unique edges ((88 × 87)/2) was generated and was made available at www.nitrc.org/projects/iit. Each element of the matrix includes the total number of streamlines connecting two gray matter labels.

#### 2.1.4. Evaluation of the white matter connectome of the IIT human brain atlas

The connectome of the IIT Human Brain Atlas v.5.0 was evaluated both qualitatively and quantitatively. First, various features of the tractogram and connectome were qualitatively compared to previously published findings. More specifically, a) probabilistic maps of transcallosal lobar connectivity were generated for the midsagittal slice of the corpus callosum, b) the thalamus was parcellated based on the strength of connectivity (i.e. highest number of streamlines) to cortical regions of different lobes, c) edge density maps of the connectome were generated (Owen et al., 2015), d) major white matter bundles (Table 1) were reconstructed from the streamlines of the tractogram using the RecoBundle approach (Garyfallidis et al., 2014; E. 2018) based on the Yeh et al., 2018 bundle models (and were made available at www.nitrc.org/projects/iit), and all output was qualitatively compared to that of previously published work.

Second, the connectome of the IIT Human Brain Atlas v.5.0 was quantitatively evaluated in terms of its spatial correspondence to those of individual young adult participants of the Human Connectome Project (HCP). For that purpose, individual tractograms and connectivity matrices were constructed for each of the 20 HCP participants of Section 2.1.2 using the same tractography approach used to construct the connectome of the atlas (the evaluation described in this section excluded connections through the optic chiasm, fornix, and medulla, in order to avoid user-bias in selecting these additional labels in each of the 20 HCP participants). The atlas connectome was filtered to remove edges with a number of streamlines lower than 5% of that of the strongest edge (Rubinov and Sporns, 2010), and the surviving 301 edges (“strong” edges) were compared between the atlas and the individual HCP connectomes in terms of spatial correspondence as follows. The streamlines of the 301 edges of each of the 21 connectomes (comprised of the atlas and 20 individual HCP connectomes) were transformed to each other connectome’s space via DTITK registration (Zhang et al., 2006) of the corresponding diffusion data (a total of 21 × 20=420 registrations). In each of the 21 connectome spaces, the volumetric overlap (OL) and volumetric overreach (OR) of streamlines were calculated between native and transformed edges, and the F_1_ score was computed to assess the spatial correspondence between native and transformed edges:

$$F_{1}\;score=2\cdot \frac{\left(1-overreach\right)\cdot overlap}{\left(1-overreach\right)+overlap},$$

using Tractometer (Côté et al., 2013) and the scripts found here https://github.com/scilus/ismrm_2015_tractography_challenge_scoring. The F_1_ score ranges from zero to one. It is zero when the overlap is zero (i.e. edges have no voxels in common), and one when the overlap is one and the overreach is zero (i.e. edges perfectly match). The Pearson’s correlation coefficient was also computed for the corresponding track-density images (TDIs) (made available at www.nitrc.org/projects/iit) to assess the similarity in track-density spatial patterns between the native and transformed edges. For each connectome, the average F_1_ scores and Pearson’s correlation coefficient over all 20 pairs of the native and transformed edges and over all edges were calculated to assess that connectome’s spatial correspondence to each other connectome. One sample *t*-test was used to test if the average F_1_ score and Pearson’s correlation for the atlas were significantly different than those for the individual HCP participants.

### 2.2. Multi-layer, connectivity-based white matter labels

**Fig. 1.**
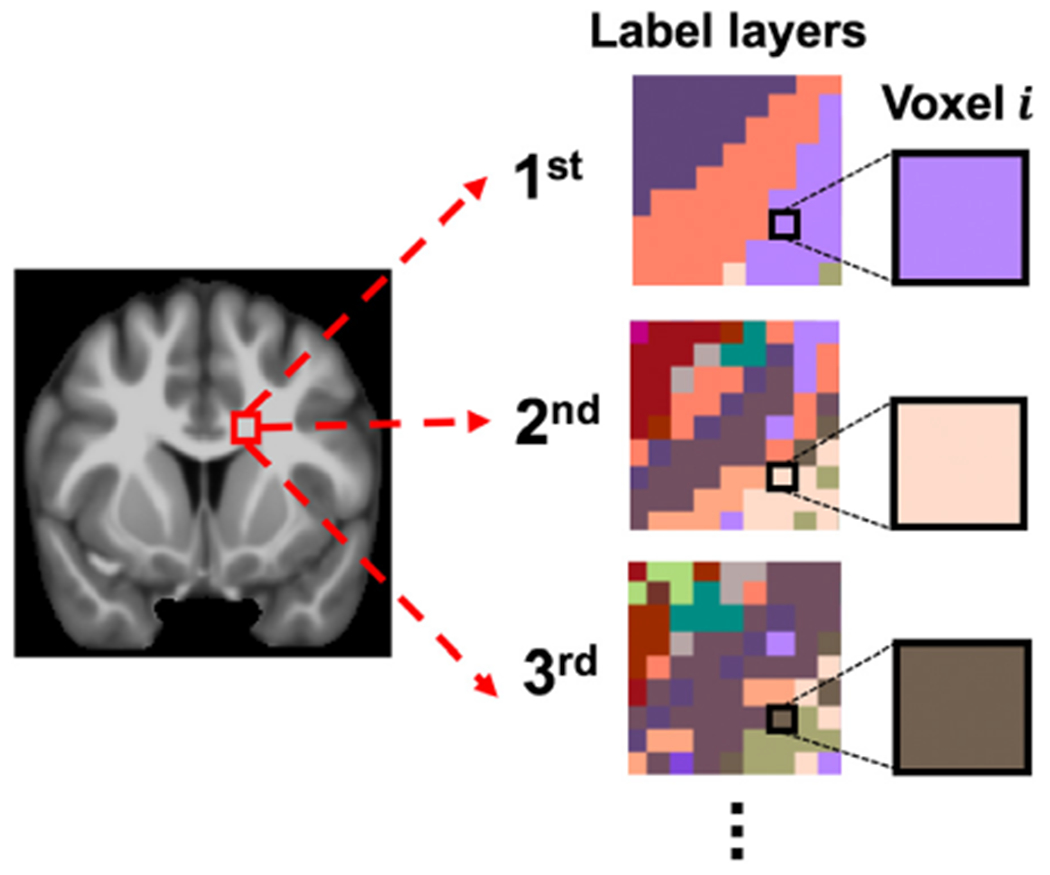
Conceptual illustration of multi-layer, connectivity-based white matter labels. Each white matter voxel is assigned a list of labels representing the most probable connections (edges) traversing that voxel in order of decreasing confidence.

A novel white matter labeling framework was introduced to describe the connectivity through white matter voxels of the atlas, and to allow the software presented in the next section to rapidly generate a list of the most probable connections traversing a white matter region. Labels that are based on connectivity information are important when it comes to white matter. In addition, fiber-crossings are very common in the brain (Jeurissen et al., 2013) and white matter voxels may contain streamlines from different edges. Consequently, assigning a unique connectivity-based label to a white matter voxel clearly misrepresents the complexity of brain connectivity. Therefore, the present work introduces the concept of multi-layer, connectivity-based labels according to which each white matter voxel is assigned a list of labels representing the most probable connections (edges) traversing that voxel (Fig. 1).

In each white matter voxel of the atlas with coordinates (*x,y,z*), the probability that a streamline passing through that voxel connects gray matter labels *i* and *j* was given by:

$$P_{i,j}\left(x,y,z\right)=\frac{S_{i,j}\left(x,y,z\right)}{\Sigma_{k=1}^{88}\Sigma_{l=1}^{88}S_{k,l}\left(x,y,z\right)}$$

where *S_i, j_* (*x, y, z*) was the number of streamlines that traverse voxel (*x,y,z*) and connect gray matter labels *i* and *j*, and $\Sigma_{k=1}^{88}\Sigma_{l=1}^{88}S_{k,l}\left(x,y,z\right)$ was the total number of streamlines that traverse that voxel and connect any pair of gray matter regions *k* and *l* (track density), according to the atlas connectome developed above. Each white matter voxel of the atlas was assigned a list of connectivity-based labels “*i,j*” (essentially edge-based labels) in order of decreasing *P _i, j_* (*x, y, z*), and a four-dimensional map of connectivity-based labels was constructed (i.e.multi-layer, connectivity-based white matter labels). A corresponding four-dimensional map containing the *P _i, j_* (*x, y, z*) for each label “*i,j*” in each voxel (*x,y,z*) of the atlas was also constructed and is referred to in the following as the white matter label confidence map. Both the multi-layer, connectivity-based labels and the corresponding confidence map were made available at www.nitrc.org/projects/iit.

### 2.3. regionconnect: rapidly extracting brain connectivity information for white matter regions in standard space

A software package named regionconnect was developed to rapidly extract standardized brain connectivity information for white matter regions with significant findings in voxel-wise neuroimaging studies, based on the atlas resources developed above. More specifically, for a white matter region in the space of the IIT atlas, regionconnect first identifies the top 60 layers of connectivity-based labels for each voxel of the region and compiles a list of all the labels across voxels (union of labels across voxels). For each label “*i,j*” in this compiled list, regionconnect calculates the probability that a streamline passing through the region connects gray matter labels *i* and *j* as follows:

$$P_{i,j}^{region}=\frac{\Sigma_{\left(x,y,z\right)}^{region}\left(P_{i,j}\left(x,y,z\right)\cdot \Sigma_{k=1}^{88}\Sigma_{l=1}^{88}S_{k,l}\left(x,y,z\right)\right)}{\Sigma_{\left(x,y,z\right)}^{region}\Sigma_{k=1}^{88}\Sigma_{l=1}^{88}S_{k,l}\left(x,y,z\right)}$$

where *P _i, j_* (*x, y, z*) is the white matter label confidence that was generated in Section 2.2 and provides the probability for label “*i,j*” in voxel (*x,y,z*), and $\Sigma_{k=1}^{88}\Sigma_{l=1}^{88}S_{k,l}\left(x,y,z\right)$ is the track density in voxel (*x,y,z*). The $\Sigma_{\left(x,y,z\right)}^{region}$ denotes summation for all white matter voxels in the region. In summary, regionconnect a) takes as input an image mask that defines the white matter region in the space of the IIT Human Brain Atlas v.5.0 (e.g. this could be a region with significant findings based on a voxel-wise analysis), b) uses information from three standardized maps in atlas space: the four-dimensional map of connectivity-based white matter labels, the corresponding label confidence map, and the three-dimensional track density map, and c) generates as output both the name and the probability of the most likely connections “*i,j*” traversing the white matter region, in order of decreasing probability.

A few additional details regarding the design, runtime, usage, and dissemination of regionconnect are provided here. The number of 60 layers of connectivity-based labels considered in each voxel of the region was empirically determined. It was shown that this number of labels per voxel was more than enough to ensure stability. Also, a) most white matter voxels (82%) had fewer than 60 labels because fewer than 60 edges had streamlines traversing those voxels, b) more than 99% of white matter voxels had fewer than 25 strong edges (edges with more streamlines than 5% of the maximum number of streamlines in a single edge), and c) a maximum of only 35 strong edges traversed the same single white matter voxel. Although there is no evidence that the number of 60 layers considered in each voxel was biologically appropriate, it was more than enough based on the available data. The time complexity of regionconnect is *O*(*k* · *n*)(~*O*(*n*)), where *k* is the number of different connections (edges) traversing a white matter region and *n* is the number of voxels in that white matter region. Regarding usage, the regionconnect tool requires that the region is either defined in the space of the IIT Human Brain Atlas v.5.0 (e.g. as a result of voxel-wise analysis conducted in that space), or is transformed to IIT space from another space. Normalization to IIT space is rather straightforward and robust for several MR and non-MR image contrasts due to the multiple available templates (www.nitrc.org/projects/iit). The accuracy achieved with the IIT Human Brain Atlas specifically for tensor-based spatial normalization has been demonstrated previously (Cabeen et al., 2017; Zhang and Arfanakis, 2018). Also regarding usage of regionconnect, in cases where voxel-wise analysis generates more than one clusters with significant findings, the user can either isolate the clusters into separate masks and run each mask through regionconnect separately to obtain separate results per cluster, or can input all clusters in the same mask to obtain results for the combination of clusters. Finally, regionconnect is available for download at www.nitrc.org/projects/iit. An online version of regionconnect in which the user is not required to load a mask but can define the region using an interactive tool is also available at www.iit.edu/~mri (best viewed on Chrome and Firefox browsers).

## 3. Results

### 3.1. White matter connectome of the IIT human brain atlas v.5.0

**Fig. 2.**
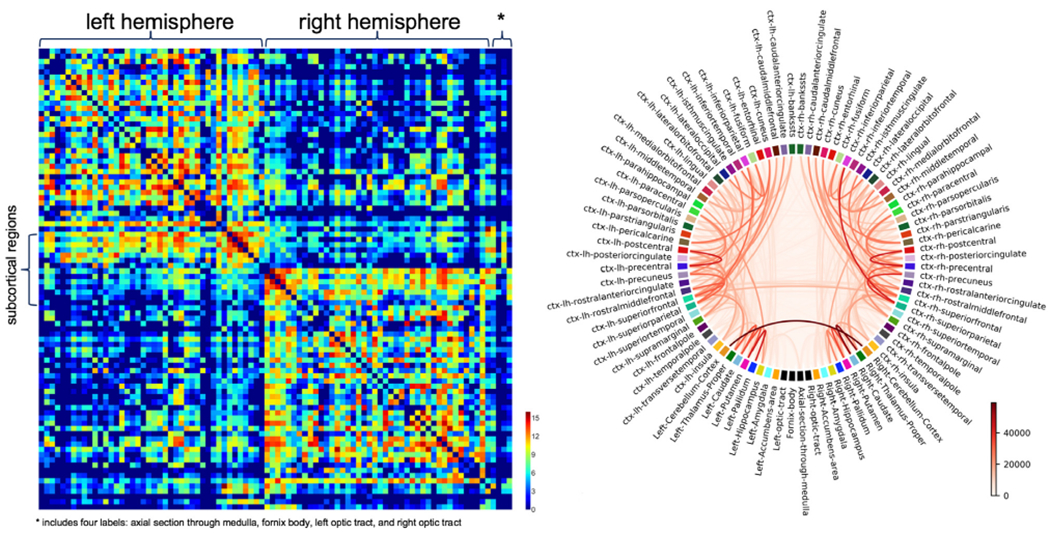
(Left) Connectivity matrix of the IIT Human Brain Atlas v.5.0. The regions corresponding to the different columns and rows are ordered as follows: left hemisphere cortical regions, left hemisphere subcortical regions, right hemisphere subcortical regions, right hemisphere cortical regions, axial section through medulla, body of the fornix, left optic tract, and right optic tract. Different colors represent different values of the base-2 logarithm of the number of streamlines connecting two regions. (Right) Circular layout of the connectivity matrix. The full names of the regions can be found in Appendix 2. The different colors of the strings represent different numbers of streamlines connecting two regions.

**Fig. 3.**
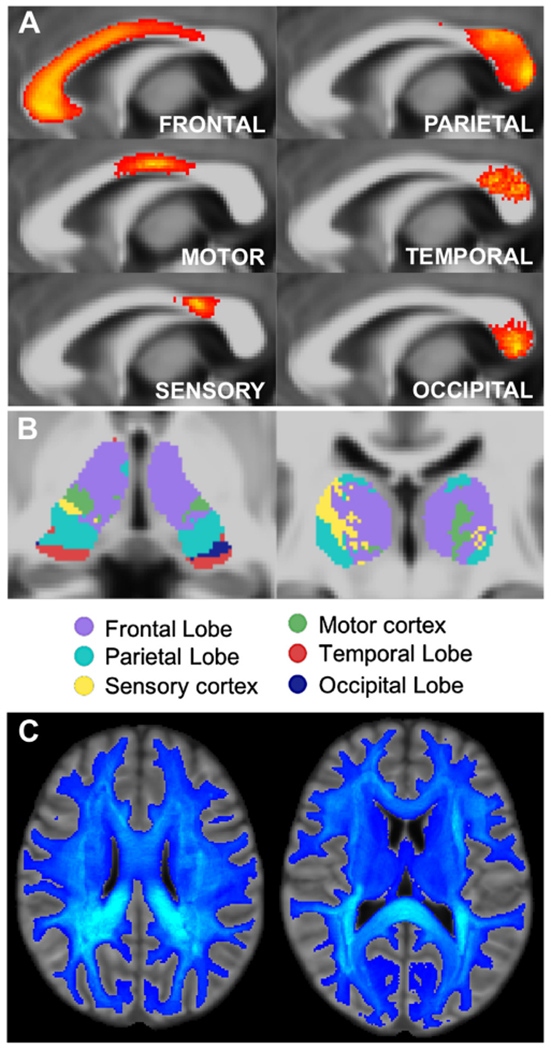
A) Probabilistic maps of transcallosal lobar connectivity for the midsagittal slice of the corpus callosum of the IIT Human Brain Atlas v.5.0. B) Connectivity-based parcellation of the thalamus using the connectome of the atlas. C) Maps of the edge density of the atlas connectome.

**Fig. 4.**
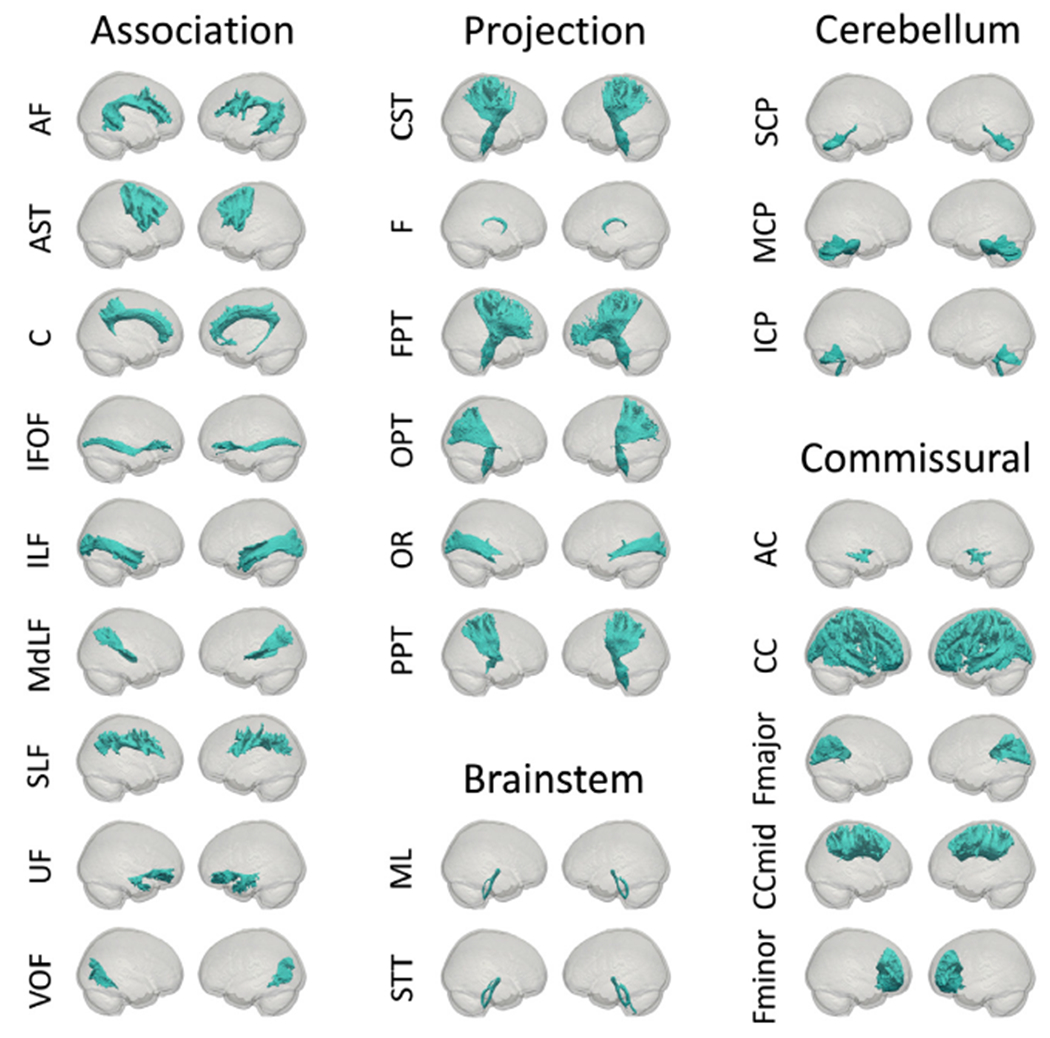
Major fiber bundles of the IIT Human Brain Atlas v.5.0.

The connectivity matrix of the new white matter connectome of the IIT Human Brain Atlas v.5.0 is shown in Fig. 2. Qualitative evaluation of the new tractogram and connectome showed first that the probabilistic maps of transcallosal lobar connectivity for the midsagittal slice of the corpus callosum of the atlas were in good agreement with the literature (Fig. 3A) (Chao et al., 2009; Park et al., 2008). Furthermore, the connectivity-based parcellation of the thalamus using the connectome of the atlas showed the expected pattern of anatomical connectivity (Fig. 3B) (Behrens et al., 2003; Morel et al., 1997). In addition, the edge density of the atlas connectome was higher in periventricular regions, in line with published work (Fig. 3C) (Owen et al., 2015). Finally, the 42 commonly used major white matter bundles (Table 1) generated based on the atlas tractogram exhibited similar structure to that seen in Yeh et al., 2018 (Fig. 4) (and were made available at www.nitrc.org/projects/iit).

**Fig. 5.**
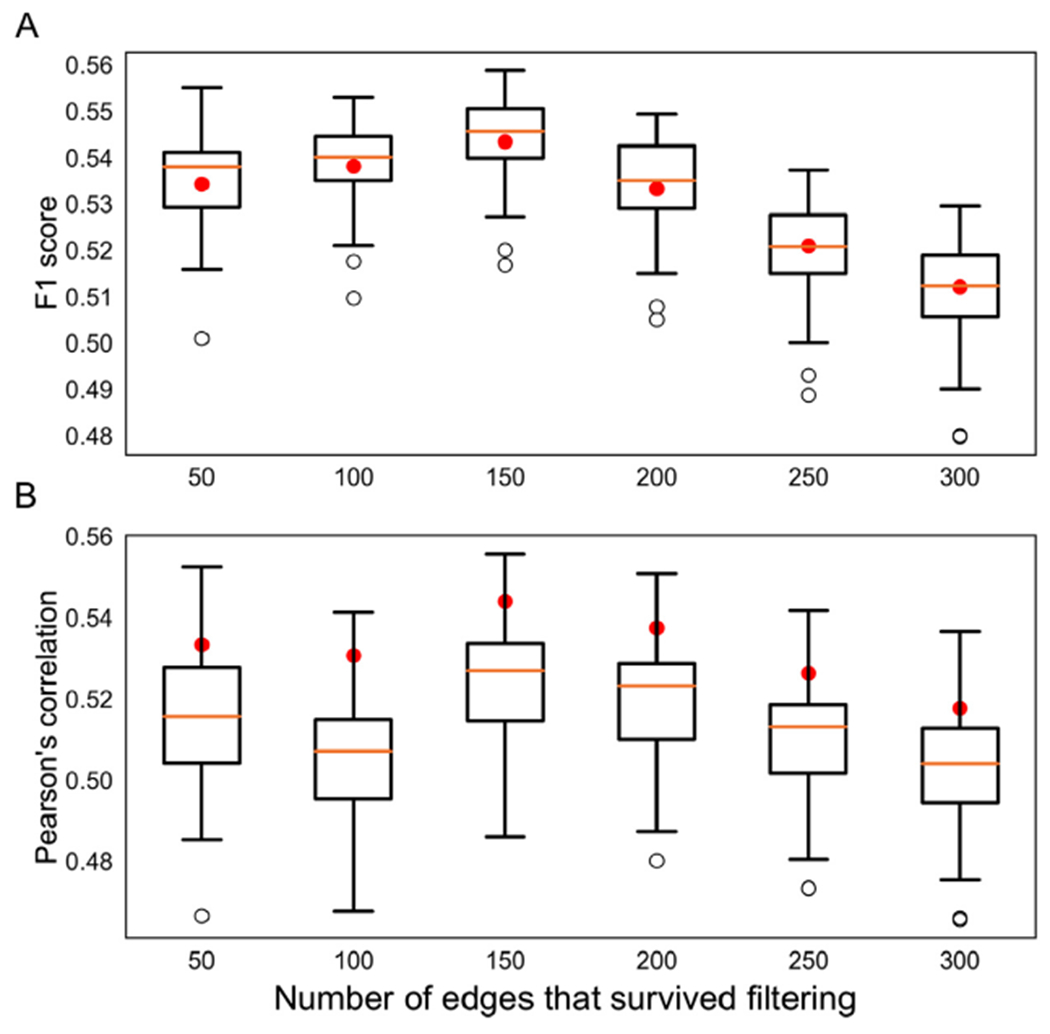
Quantitative assessment of the spatial correspondence between the edges of the connectome of the IIT Human Brain Atlas v.5.0 and the edges of 20 individual HCP participants. The box-plots present the A) average F1 score and B) average Pearson’s correlation of track density maps over all pairs of native and spatially transformed edges and over all edges when an HCP participant is used as a reference. The red dots correspond to the average F1 score or average Pearson’s correlation when the atlas is used as a reference. Results are shown for different levels of edge filtration (50–300 edges surviving filtering).

Quantitative assessment of the spatial correspondence between the connectome of the IIT Human Brain Atlas v.5.0 and those of individual HCP participants demonstrated that the average F_1_ score when the atlas is used as reference was not significantly different than the mean of the average F_1_ scores when each HCP participant was used as a reference (Fig. 5A). This suggests that the spatial organization of the streamlines in the edges of the IIT connectome is representative of that of individual HCP connectomes. In addition, the average Pearson’s correlation of the track density maps when the atlas was used as a reference was significantly higher than the mean of the average Pearson’s correlations when each HCP participant was used as a reference (*p* < 0.0001) (Fig. 5B). This indicates that the spatial patterns of track-density of the atlas connectome are more similar on average to those of individual HCP connectomes compared to the similarity of one HCP connectome to another. These quantitative results were consistent for different amounts of edge filtration (considering at minimum the 50 strongest edges and at maximum the 301 strongest edges).

### 3.2. Multi-layer, connectivity-based white matter labels

**Fig. 6.**
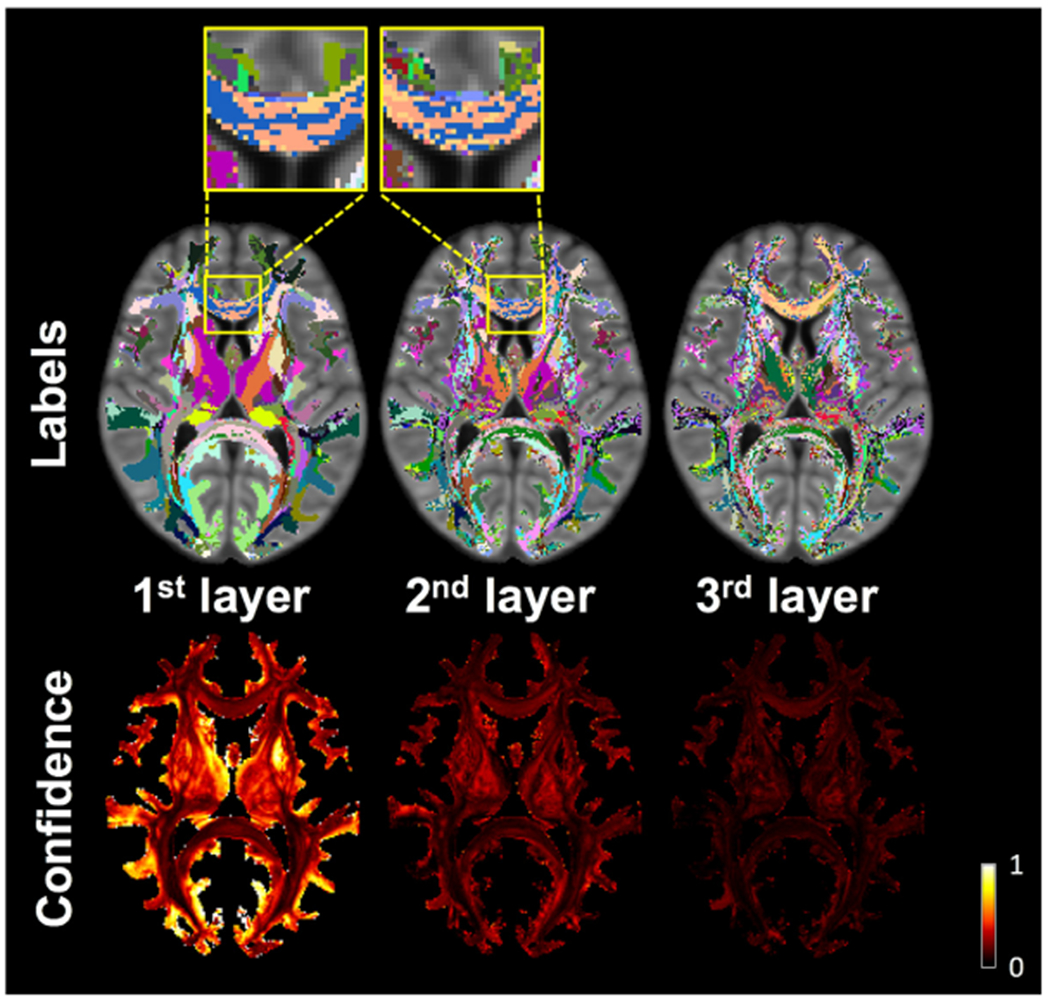
Top three layers of white matter labels for a single axial slice of the IIT Human Brain Atlas v.5.0 and corresponding confidence maps.

Maps of the top 3 layers of the multi-layer, connectivity-based white matter labels of the IIT Human Brain Atlas v.5.0 and the corresponding confidence maps are shown in Fig. 6. The labels of the top layer represent the most likely connection through each voxel and form clusters that are rather symmetric across hemispheres. The maps of subsequent layers of labels appear less organized since e.g. the 3rd label in one voxel (3rd most likely connection) may be the 4th label (4th most likely connection) in the neighboring voxel. Confidence maps show highest probabilities for labels of the top layer and decreasing probabilities for subsequent layers. White matter voxels dominated by a single connection have substantially higher confidence in the top layer compared to subsequent layers. In contrast, white matter voxels that don’t have a single dominating connection through them, have a low confidence in the top layer and relatively similar confidence in more than one layers. A clear demonstration of the need for multi-layer labels in white matter is provided in the magnified view of the top two layers of labels in the anterior portion of the corpus callosum in Fig. 6. The top two layers of those voxels contain two labels (colored blue and orange in Fig. 6), the order of which is swapped across voxels, and their confidence values are similar. This suggests that there are at least two equally important connections in these voxels that have only minor differences in probability of occurrence. It is therefore imperative to provide multi-layer labels in such voxels instead of a single label. The same observation (i.e. neighboring voxels having alternating top layer and second or third layer labels with similar confidence) can be made in several other parts of the white matter.

### 3.3 regionconnect

**Fig. 7.**
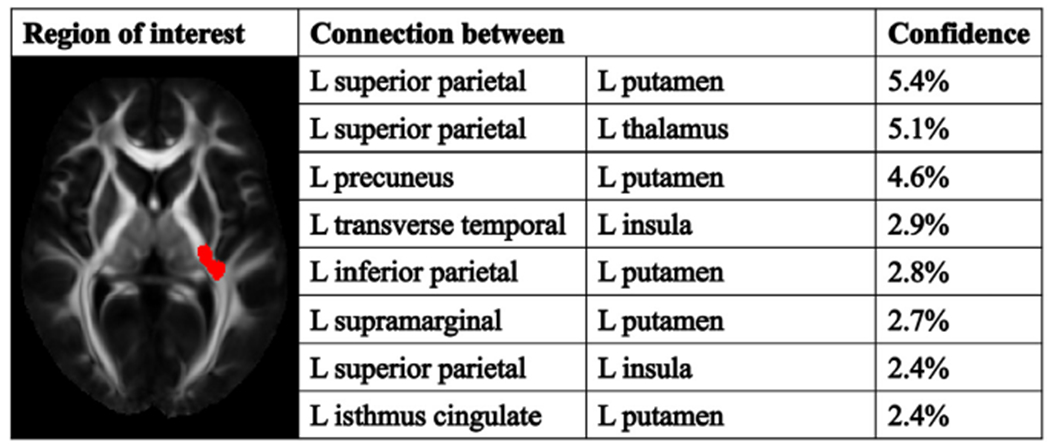
Output of regionconnect for the 500 mm^3^ region of interest shown in red. The table shows the eight most likely connections traversing the region of interest and the corresponding confidence for each connection.

Testing regionconnect in single white matter voxels and regions of different sizes provided brain connectivity information rapidly and reproducibly. Fig. 7 shows an example of a 500 mm^3^ white matter region with significant findings in a simulated voxel-wise analysis conducted in the space of the IIT Human Brain Atlas v.5.0, and a list of the most probable connections through that region provided by regionconnect. The runtime for this region was 13.7 s on a single processor of an average personal computer. In the case of multiple clusters of voxels showing significant findings in a voxel-wise analysis, regionconnect can be run per cluster or per group of clusters. All results were fully reproducible since regionconnect is based on standardized connectivity information.

## 4. Discussion

The present work developed an approach named regionconnect for rapidly extracting standardized brain connectivity information for white matter regions with significant findings in voxel-wise neuroimaging studies. This approach addresses a long-standing lack of specificity in terms of brain connectivity when reporting statistically significant findings in white matter. The new approach does not require diffusion MRI data with angular resolution that is sufficiently high for tractography, in fact it does not require diffusion MRI or any MRI data, and can be used even in populations where tractography is not warranted due to certain brain lesions. It is based on the healthy young adult brain connectome that has been precalculated for the IIT atlas and has been stored in multi-layer, connectivity-based labels allowing fast retrieval and integration.

The information generated by regionconnect is standardized and represents the average healthy young adult brain connectivity. Regionconnect provides a list of the most likely adult brain connections that would normally traverse a white matter region, and does not reflect the actual connectivity of the population under investigation. Regionconnect produces the same output for the same region independent of the population under study, and does not capture interindividual variations that may have been averaged-out in the atlas. In fact, regionconnect provides the same level of information for linking white matter findings to brain connectivity as that provided by gray matter resources for linking gray matter findings to brain function. For example, providing standard space coordinates of a gray matter region allows use of a brain function database to infer function, but it is not necessary that the function supported by that gray matter region is the same for the population under study or for every individual in that population. In addition, regionconnect is superior to a static database or lookup table since it can dynamically integrate information for any combination of voxels.

Regionconnect is based on the white matter connectome that was developed in this work for the IIT Human Brain Atlas v.5.0. Qualitative and quantitative evaluation of the new tractogram and connectome showed that the new resources contain connectivity information that is in line with the literature. It was also demonstrated that the spatial organization of streamlines in the edges of the atlas connectome is representative of that of individual HCP connectomes. Furthermore, it was shown that the spatial patterns of track-density of the atlas are more similar on average to those of individual HCP connectomes compared to the similarity across individual HCP connectomes, which may be due to the substantially lower noise in the atlas dataset compared to singlesubject HCP datasets. Thus, it can be concluded that regionconnect is based on high quality brain connectivity resources. Nevertheless, in the core of all the above resources is tractography which has known limitations (Jones, 2010; Jones et al., 2013; Maier-Hein et al., 2017), hence the information generated by regionconnect constitutes only estimates of the average healthy young adult brain connectivity and not the true connectivity.

The concept of multi-layer, connectivity-based white matter labels was also introduced in this work. The significance of this new white matter labeling framework is twofold. First, it captures connectivity information and therefore directly characterizes the main role of white matter in the brain. Second, it allows multiple labels per white matter voxel which is consistent with the fact that each voxel may contain axons from multiple connections (Jeurissen et al., 2013). It was demonstrated that certain white matter voxels include one dominating connection, while others include a number of connections that are almost equally probable. Thus, it can be concluded that compared to any other white matter labeling approach published to date, the multi-layer, connectivity-based white matter labels introduced here may best capture the main role of white matter.

The resources that were generated for the IIT atlas allow the user to define new multi-layer, connectivity-based labels in order to best fit the research question at hand. The level of detail in the connectivity information provided through multi-layer labels can be adjusted by using a gray matter parcellation scheme that is finer or coarser than that used here. For example, by combining the IIT tractogram with the smaller Destrieux gray matter regions (also available in IIT space) (Destrieux et al., 2010) instead of the Desikan regions used in this work, one can increase the level of connectivity detail. In contrast, by combining multiple gray matter regions to define the cortex per lobe, one can lower the level of detail and generate multi-layer white matter labels containing lobar connectivity information. Similarly, one can alter the type of connectivity information included in the multi-layer white matter labels by using a gray matter parcellation scheme that is based on a different characteristic of gray matter (e.g. cytoarchitecture or function) than that used here (i.e. gyral-based Freesurfer regions).

By efficiently storing connectivity information per voxel, the multi-layer labels allow regionconnect to integrate information across voxels rapidly. The only other processing step that may be required when using regionconnect in voxel-wise investigations is transformation of the regions with significant findings to IIT space, which can be completed fast, and may not even be necessary if the IIT space is selected for the voxel-wise analysis. Thus, applying regionconnect in voxel-wise studies may enhance specificity in terms of brain connectivity, with only a negligible amount of added processing time.

Regionconnect and other resources developed in this work have already been applied successfully in healthy young adults as well as other populations. Regionconnect was recently used in voxel-wise investigations involving diffusion tensor imaging data on older adults (Han et al., 2016; 2018; Lamar et al., 2019), and also in a study of aging with ex-vivo MRI but no diffusion data (Gaiteri et al., 2019). The online interactive version of regionconnect that allows the user to easily select and update a white matter region of interest has been used in education for exploring healthy adult brain connectivity. The tractogram can be used to identify streamlines that traverse a white matter region of interest, and this was done recently in older adults with progranulin-associated frontotemporal dementia to determine streamlines affected by white matter hyperintensities (Sudre et al., 2019). The tractogram can also be used to visualize the group of streamlines connecting two gray matter regions. For example, after regionconnect reports the most likely connections traversing a white matter region, the corresponding streamlines of those connections can be selected and visualized. The track-density images corresponding to the different edges of the connectome have been used towards whole brain network analyses. More specifically, the track-density images can be converted to masks (one mask per edge), transformed to the space of individual subjects, and used to extract average values of different MRI properties per edge, which can then be used in network analyses. This was done recently in adults with moderate to severe traumatic brain injury (Jolly et al., 2020). Similarly, the major bundles derived from the IIT connectome have also been used as masks for region of interest analyses. Examples of such application include a study in adolescents with anorexia nervosa (Breithaupt et al., 2020), investigations in healthy children (Kocevar et al., 2019) and adults (Tarun et al., 2020), a large multi-site diffusion MRI study of schizophrenia (Cetin-Karayumak et al., 2019), and a project that developed an approach for harmonization of multi-site diffusion MRI data (Cetin-Karayumak et al., 2019). Since the accuracy of regionconnect directly depends on the accuracy of registration to the IIT space, it should also be noted that registration to the IIT diffusion tensor template has been shown to allow higher spatial normalization accuracy compared to all other available tensor templates, including study-specific templates constructed with state of the art techniques, for both younger and older adults (Cabeen et al., 2017; Zhang and Arfanakis, 2018). The diffusion tensor template of the atlas was successfully used for spatial normalization in a study of the effects of tetrahydrocannabinol on the brain of young adults (Cabeen et al., 2020), a study of diffuse axonal injury (Ware et al., 2017), an investigation of the relation between fructose intake and brain connectivity in children (Clark et al., 2020), a study on the effects of perivascular spaces on water diffusion in white matter (Sepehrband et al., 2019), and studies that generated atlases of brainstem nuclei in IIT space (Singh et al., 2019) (Garcia-Gomar et al., 2019). The HARDI template of the atlas, which served as the foundation for the tractogram developed in the present work, was recently used to perform whole brain tractography in studies of depression (Avecillas-Chasin et al., 2019), white matter maturation from infancy to adolescence (Lynch et al., 2020), and Parkinson’s disease progression (Yau et al., 2018). Finally, the T1-weighted template and gray matter labels of the IIT atlas that defined the gray matter nodes in the present work, were used for region of interest analyses in children and young adults with Down syndrome (Lee et al., 2020), as well as for facilitating common edges across subjects in studies of amyloid in brain connectivity networks and studies on the association of CSF biomarkers with decline in white matter connectivity in cognitively unimpaired middle-aged and older adults (Hwang et al., 2019) (Kim et al., 2019). Overall, the above demonstrate that regionconnect and other IIT atlas resources developed or used in this work have been applied successfully in health and disease and in various age-groups.

The main limitation of regionconnect, which was also discussed earlier in this section, is the fact that it is based on tractography and thus provides only estimates, and not the true, average healthy young adult brain connectivity. Nonetheless, regionconnect is based on a tractogram generated using high quality atlas data and state of the art tractography methods, and was shown to include connectivity information that is in agreement with the literature and representative of HCP connectivity. Also, regionconnect does not capture interindividual variations that may have been averaged-out in the atlas. Another limitation of regionconnect is that regions with significant findings in voxel-wise investigations may need to be transformed to IIT space if the original voxel-wise analysis is not conducted in IIT space. However, this step can be completed accurately and fast using anatomy-based, tensor-based, or fODF-based registration to the corresponding templates of the atlas. Regionconnect may not be appropriate in studies of persons with large lesions, or in studies with very low image quality (e.g. very low spatial resolution or contrast, or very high noise or artifact content), due to potential registration failures. However, this limitation may not be worth considering since such data would probably not be used in voxel-wise analyses exactly due to the potential for misregistration.

## 5. Conclusion

An approach named regionconnect was developed for rapidly extracting standardized brain connectivity information for white matter regions with significant findings in voxel-wise neuroimaging studies. This approach addresses a long-standing lack of specificity in terms of brain connectivity when reporting statistically significant findings in white matter. Regionconnect is based on the healthy young adult brain connectome that was calculated for the IIT atlas and was stored in multi-layer, connectivity-based labels allowing fast retrieval and integration. Regionconnect does not require high angular resolution diffusion MRI or any MRI data, and can be used even in populations with certain brain lesions. The regionconnect software as well as the white matter tractogram, major fiber-bundles, connectome, track density images of the corresponding edges, multi-layer connectivity-based labels, and other resources developed in this work for the IIT Human Brain Atlas v.5.0 were made available at www.nitrc.org/projects/iit. An interactive, online version of regionconnect is available at www.iit.edu/~mri.

## Supplementary Material

1

2

## Figures and Tables

**Table 1:** List of the 42 white matter bundles reconstructed from the tractogram of the IIT Human Brain Atlas.

White Matter Bundle Abbreviations	White Matter Bundle Names
AC	Anterior Commissure
AF_L	Left Arcuate Fasciculus
AF_R	Right Arcuate Fasciculus
AST_L	Left Frontal Aslant Tract
AST_R	Right Frontal Aslant Tract
C_L	Left Cingulum
C_R	Right Cingulum
Fmajor	Forceps Major
Fminor	Forceps Minor
CC	Corpus Callosum
CCmid	Middle Of Corpus Callosum
CST_L	Left Corticospinal Tract
CST_R	Right Corticospinal Tract
F	Fornix
FPT_L	Left Frontopontine
FPT_R	Right Frontopontine
ICP_L	Left Inferior Cerebellar Peduncle
ICP_R	Right Inferior Cerebellar Peduncle
IFOF_L	Left Inferior Frontooccipital Fasciculus
IFOF_R	Right Inferior Frontooccipital Fasciculus
ILF_L	Left Inferior Longitudinal Fasciculus
ILF_R	Right Inferior Longitudinal Fasciculus
MCP	Middle Cerebellar Peduncle
MdLF_L	Left Middle Longitudinal Fasciculus
MdLF_R	Right Middle Longitudinal Fasciculus
ML_L	Left Medial Lemniscus
ML_R	Right Medial Lemniscus
OPT_L	Left Occipitopontine Tract
OPT_R	Right Occipitopontine Tract
OR_L	Left Optic Radiation
OR_R	Right Optic Radiation
PPT_L	Left Parietopontine Tract
PPT_R	Right Parietopontine Tract
SCP	Superior Cerebellar Peduncle
SLF_L	Left Superior Longitudinal Fasciculus
SLF_R	Right Superior Longitudinal Fasciculus
STT_L	Left Spinothalamic Tract
STT_R	Right Spinothalamic Tract
UF_L	Left Uncinate Fasciculus
UF_R	Right Uncinate Fasciculus
VOF_L	Left Vertical Occipital Fasciculus
VOF_R	Right Vertical Occipital Fasciculus
