# Supplementary material for: Regionconnect: Rapidly extracting standardized brain connectivity information in voxel-wise neuroimaging studies
Source: Neuroimage. Author manuscript; Available in PMC 2021 Jan 17. (PMC7811895; doi:10.1016/j.neuroimage.2020.117462)
Supplement: 1 [file NIHMS1659128-supplement-1.docx]

**Appendix 1.**

The tractography approach used in this work was evaluated using Tractometer and the framework described in Maier-Hein et al., 2017. The evaluation was conducted on the artifact-free data. In summary, the number of valid bundles (VB) was 24 and the number of invalid bundles (IB) was 94. The mean overlap (OL) value was 76.0% and the mean overreach (OR) value was 30.4% (considering the CP bundle in the calculation of the means). Valid connection (VC), invalid connection (IC) and non-connection (NC) ratios were 46.1 %, 29.1% and 24.8%, respectively (note that the VC, IC and NC metrics are not compatible with anatomically constrained tractography (ACT) used in this work as discussed in Maier-Hein et al., 2017, Supplementary Figure 4). The following table provides detailed Tractometer results per bundle.

| **Bundle name** | **Number of streamlines** | **Overlap (OL) (%)** | **Overreach (OR) (%)** | **F_1_ score (%)** |
| --- | --- | --- | --- | --- |
| ICP_left | 5069 | 80.0 | 38.9 | 69.3 |
| FPT_right | 11061 | 90.3 | 36.8 | 74.4 |
| CST_left | 2541 | 83.9 | 36.7 | 72.2 |
| Cingulum_left | 7347 | 74.1 | 27.2 | 73.4 |
| OR_right | 2260 | 80.1 | 33.7 | 72.6 |
| ICP_right | 2964 | 75.3 | 34.8 | 69.9 |
| Fornix | 644 | 59.7 | 28.2 | 65.2 |
| SLF_right | 15368 | 87.8 | 33.3 | 75.5 |
| UF_right | 5985 | 91.7 | 32.9 | 77.5 |
| CA | 1 | 11.3 | 9.7 | 20.1 |
| SCP_right | 612 | 66.3 | 33.0 | 66.6 |
| CC | 83355 | 88.9 | 27.1 | 80.1 |
| POPT_right | 2359 | 91.7 | 37.2 | 74.5 |
| CST_right | 2726 | 77.0 | 29.5 | 73.6 |
| UF_left | 7333 | 92.9 | 28.2 | 81.0 |
| ILF_right | 14386 | 88.3 | 43.9 | 68.6 |
| SCP_left | 620 | 70.9 | 23.3 | 73.7 |
| CP | 0 | 0 | 0 | 0 |
| POPT_left | 2641 | 87.2 | 30.3 | 77.4 |
| FPT_left | 5020 | 80.7 | 49.1 | 62.4 |
| ILF_left | 15551 | 89.9 | 36.5 | 74.4 |
| OR_left | 1545 | 83.4 | 39.2 | 70.4 |
| SLF_left | 16557 | 87.4 | 37.3 | 73.0 |
| Cingulum_right | 8157 | 72.5 | 18.1 | 76.9 |
| MCP | 16365 | 87.8 | 15.5 | 86.1 |
