# Supplementary material for: Regionconnect: Rapidly extracting standardized brain connectivity information in voxel-wise neuroimaging studies
Source: Neuroimage. Author manuscript; Available in PMC 2021 Jan 17. (PMC7811895; doi:10.1016/j.neuroimage.2020.117462)
Supplement: 2 [file NIHMS1659128-supplement-2.docx]

**Appendix 2.**

Augmented list of gray matter labels used in the development of the connectome of the IIT Human Brain Atlas v.5.0.

| **Label ID** | **Abbreviations** | **Regions** |
| --- | --- | --- |
| 1 | ctx-lh-bankssts | Banks of the superior temporal sulcus (left hemisphere) |
| 2 | ctx-lh-caudalanteriorcingulate | Caudal anterior cingulate cortex (left hemisphere) |
| 3 | ctx-lh-caudalmiddlefrontal | Caudal middle frontal gyrus (left hemisphere) |
| 4 | ctx-lh-cuneus | Cuneus (left hemisphere) |
| 5 | ctx-lh-entorhinal | Entorhinal cortex (left hemisphere) |
| 6 | ctx-lh-fusiform | Fusiform gyrus (left hemisphere) |
| 7 | ctx-lh-inferiorparietal | Inferior parietal cortex (left hemisphere) |
| 8 | ctx-lh-inferiortemporal | Inferior temporal gyrus (left hemisphere) |
| 9 | ctx-lh-isthmuscingulate | Isthmus cingulate cortex (left hemisphere) |
| 10 | ctx-lh-lateraloccipital | Lateral occipital cortex (left hemisphere) |
| 11 | ctx-lh-lateralorbitofrontal | Lateral orbitofrontal cortex (left hemisphere) |
| 12 | ctx-lh-lingual | Lingual gyrus (left hemisphere) |
| 13 | ctx-lh-medialorbitofrontal | Medial orbitofrontal cortex (left hemisphere) |
| 14 | ctx-lh-middletemporal | Middle temporal gyrus (left hemisphere) |
| 15 | ctx-lh-parahippocampal | Parahippocampal gyrus (left hemisphere) |
| 16 | ctx-lh-paracentral | Paracentral lobule (left hemisphere) |
| 17 | ctx-lh-parsopercularis | Pars opercularis (left hemisphere) |
| 18 | ctx-lh-parsorbitalis | Pars orbitalis (left hemisphere) |
| 19 | ctx-lh-parstriangularis | Pars triangularis (left hemisphere) |
| 20 | ctx-lh-pericalcarine | Pericalcarine cortex (left hemisphere) |
| 21 | ctx-lh-postcentral | Postcentral gyrus (left hemisphere) |
| 22 | ctx-lh-posteriorcingulate | Posterior cingulate cortex (left hemisphere) |
| 23 | ctx-lh-precentral | Precentral gyrus (left hemisphere) |
| 24 | ctx-lh-precuneus | Precuneus cortex (left hemisphere) |
| 25 | ctx-lh-rostralanteriorcingulate | Rostral anterior cingulate cortex (left hemisphere) |
| 26 | ctx-lh-rostralmiddlefrontal | Rostral middle frontal gyrus (left hemisphere) |
| 27 | ctx-lh-superiorfrontal | Superior frontal gyrus (left hemisphere) |
| 28 | ctx-lh-superiorparietal | Superior parietal cortex (left hemisphere) |
| 29 | ctx-lh-superiortemporal | Superior temporal gyrus (left hemisphere) |
| 30 | ctx-lh-supramarginal | Supramarginal gyrus (left hemisphere) |
| 31 | ctx-lh-frontalpole | Frontal pole (left hemisphere) |
| 32 | ctx-lh-temporalpole | Temporal pole (left hemisphere) |
| 33 | ctx-lh-transversetemporal | Transverse temporal cortex (left hemisphere) |
| 34 | ctx-lh-insula | Insula (left hemisphere) |
| 35 | Left-Cerebellum-Cortex | Left Cerebellum (cortex) |
| 36 | Left-Thalamus-Proper | Left Thalamus Proper |
| 37 | Left-Caudate | Left Caudate |
| 38 | Left-Putamen | Left Putamen |
| 39 | Left-Pallidum | Left Pallidum |
| 40 | Left-Hippocampus | Left Hippocampus |
| 41 | Left-Amygdala | Left Amygdala |
| 42 | Left-Accumbens-area | Left Accumbens area |
| 43 | Right-Thalamus-Proper | Right Thalamus Proper |
| 44 | Right-Caudate | Right Caudate |
| 45 | Right-Putamen | Right Putamen |
| 46 | Right-Pallidum | Right Pallidum |
| 47 | Right-Hippocampus | Right Hippocampus |
| 48 | Right-Amygdala | Right Amygdala |
| 49 | Right-Accumbens-area | Right Accumbens area |
| 50 | ctx-rh-bankssts | Banks of the superior temporal sulcus (right hemisphere) |
| 51 | ctx-rh-caudalanteriorcingulate | Caudal anterior cingulate cortex (right hemisphere) |
| 52 | ctx-rh-caudalmiddlefrontal | Caudal middle frontal gyrus (right hemisphere) |
| 53 | ctx-rh-cuneus | Cuneus (right hemisphere) |
| 54 | ctx-rh-entorhinal | Entorhinal cortex (right hemisphere) |
| 55 | ctx-rh-fusiform | Fusiform gyrus (right hemisphere) |
| 56 | ctx-rh-inferiorparietal | Inferior parietal cortex (right hemisphere) |
| 57 | ctx-rh-inferiortemporal | Inferior temporal gyrus (right hemisphere) |
| 58 | ctx-rh-isthmuscingulate | Isthmus cingulate cortex (right hemisphere) |
| 59 | ctx-rh-lateraloccipital | Lateral occipital cortex (right hemisphere) |
| 60 | ctx-rh-lateralorbitofrontal | Lateral orbitofrontal cortex (right hemisphere) |
| 61 | ctx-rh-lingual | Lingual gyrus (right hemisphere) |
| 62 | ctx-rh-medialorbitofrontal | Medial orbitofrontal cortex (right hemisphere) |
| 63 | ctx-rh-middletemporal | Middle temporal gyrus (right hemisphere) |
| 64 | ctx-rh-parahippocampal | Parahippocampal gyrus (right hemisphere) |
| 65 | ctx-rh-paracentral | Paracentral lobule (right hemisphere) |
| 66 | ctx-rh-parsopercularis | Pars opercularis (right hemisphere) |
| 67 | ctx-rh-parsorbitalis | Pars orbitalis (right hemisphere) |
| 68 | ctx-rh-parstriangularis | Pars triangularis (right hemisphere) |
| 69 | ctx-rh-pericalcarine | Pericalcarine cortex (right hemisphere) |
| 70 | ctx-rh-postcentral | Postcentral gyrus (right hemisphere) |
| 71 | ctx-rh-posteriorcingulate | Posterior cingulate cortex (right hemisphere) |
| 72 | ctx-rh-precentral | Precentral gyrus (right hemisphere) |
| 73 | ctx-rh-precuneus | Precuneus cortex (right hemisphere) |
| 74 | ctx-rh-rostralanteriorcingulate | Rostral anterior cingulate cortex (right hemisphere) |
| 75 | ctx-rh-rostralmiddlefrontal | Rostral middle frontal gyrus (right hemisphere) |
| 76 | ctx-rh-superiorfrontal | Superior frontal gyrus (right hemisphere) |
| 77 | ctx-rh-superiorparietal | Superior parietal cortex (right hemisphere) |
| 78 | ctx-rh-superiortemporal | Superior temporal gyrus (right hemisphere) |
| 79 | ctx-rh-supramarginal | Supramarginal gyrus (right hemisphere) |
| 80 | ctx-rh-frontalpole | Frontal pole (right hemisphere) |
| 81 | ctx-rh-temporalpole | Temporal pole (right hemisphere) |
| 82 | ctx-rh-transversetemporal | Transverse temporal cortex (right hemisphere) |
| 83 | ctx-rh-insula | Insula (right hemisphere) |
| 84 | Right-Cerebellum-Cortex | Right Cerebellum (cortex) |
| 85 | Axial-section-through-medulla | Axial section through medulla |
| 86 | Fornix-body | Fornix body |
| 87 | Left-optic-tract | Left optic tract |
| 88 | Right-optic-tract | Right optic tract |
